# Positive Influence of Cooperative Learning and Emotion Regulation on EFL Learners’ Foreign Language Enjoyment

**DOI:** 10.3390/ijerph191912604

**Published:** 2022-10-02

**Authors:** Songyun Zheng, Xiang Zhou

**Affiliations:** College of Foreign Languages, Shanghai Maritime University, Shanghai 201306, China; zhengsy@shmtu.edu.cn

**Keywords:** foreign language enjoyment, cooperative learning, positive goal interdependence, personal peer support, emotion regulation

## Abstract

This study approaches foreign language enjoyment (FLE) through the lenses of positive psychology, and in particular, examines how enjoyment is affected by emotion regulation (ER) and two factors concerning cooperative learning (CL) in a classroom climate, namely positive goal interdependence (PGI) and peer personal support (PPS). To achieve this goal, 115 Chinese university freshmen (male 47; female 68) aged between 18 to 20 (M = 18.69; SD = 0.65) were invited to complete a questionnaire. Regression analyses revealed a clear three-factor structure determining the FLE of students learning English as a foreign language (EFL), which are ER, PGI that highlights cooperation, and PPS that emphasizes the interpersonal relationship between peers. It also showed that PGI and PPS significantly influence each other while positively and jointly shaping FLE. The findings suggest that university EFL students with higher ER abilities are more likely to obtain enjoyment in the learning process and that positive interdependence and interpersonal support during CL also play an effective role in deciding students’ FLE. The study not only confirms the importance of ER and CL which may lead to high-level learning enjoyment, but also provides practical implications for the realization of an enjoyable second language acquisition (SLA) experience.

## 1. Introduction

The role of emotions in an educational context is crucial. That is why the study of FLE, which has marked the “positive renaissance” in SLA research since 2012 [1], serves as a significant aspect of illuminating foreign language learners’ emotions [2]. Researchers, with various cultural backgrounds, examined samples from around the world [3] and have been paying attention to foreign language enjoyment (FLE) online and offline [4,5] which can affect language learners’ well-being as well as their interest in foreign languages (FL) [6,7]. Emotions of EFL students simultaneously have been receiving close academic attention from scholars [8,9], and many researchers have approached students’ emotions within the theoretical framework of positive psychology (PP) [10]. The reasons for approaching students’ emotions from a PP perspective is because their performance in various educational contexts are not only determined by their learning ability, but also their capability of managing and regulating emotions during the learning process [11]. Despite the fact that various factors associated with FLE have been investigated, what seems to be under-explored is the shaping factors of FLE in a CL context.

Similar to emotions, cooperative learning (CL) has been proven to play a pivotal role in both education and psychology [12]. Studies on Chinese EFL students’ CL have been carried out to explore the significance of cooperation in shaping learners’ cooperative autonomy [13] and their improvements in self-learning capabilities and language skills [14]. Furthermore, the mediating role of peer factors [15] as well as cultural and technological factors have been proven to play an active role in shaping students’ CL performance [16]. What needs to be further investigated, on top of FLE’s relations with negative emotions such as burnout [17], anxiety [18,19], and boredom [20], is how CL, especially which aspects of cooperation, can shape students’ FLE. Existing research, however, rarely specifically investigated the impact of PPS and PGI, two positive elements concerning CL, on university EFL students’ FLE from a PP perspective. Although the impact of teachers, learner autonomy, and social experiences have been investigated in order to illuminate FLE [21], the significance of CL, which emphasizes interpersonal relationships in a classroom climate as well as its role in shaping FLE, has not received adequate attention.

Moreover, if enjoyment and anxiety are regarded as the left and right feet of learners [22], the coordination of both need to involve emotion regulation (ER) [23] and how students can boost their enjoyment during the FL learning process.

Based on what has been discussed above, this study, with the aim of seeking a better learning experience, specifically focused on how students could best make use of ER and positive cooperation to attain positive attitudes towards FL and achieve FLE. It thus sheds insightful light on the relationship between CL, ER, and FLE, which so far has rarely been explored. The study may serve as a useful reference for both teachers and students who are part and parcel of the university’s activities concerning EFL teaching and learning. In the following part of this section, a review of relevant scholarship regarding EFL learners’ CL, emotions, FLE, and ER will be provided, followed by an elucidation of the research hypotheses and the key terms used in this study.

### 1.1. Overview of the Literature

EFL students’ CL: In an educational context, CL has long been regarded as a determining factor which shapes the classroom climate [24] and has been proven to play an effective role in determining students’ learning motivation [25] and in improving their language skills, such as speaking [26], reading [27], and academic writing [28]. Nowadays, whether online or offline, CL can further be realized in various forms with the assistance of information technology. In the process of conducting CL, what cannot be ignored is that various emotions may be experienced by students, including social and technology anxiety [29] as well as enjoyment [30]. What is worth examining but seems to be under-researched is which factors can shape students’ emotions, especially their FLE, in a CL context.

Emotions in education and FLE: Whether inside or outside the classroom, the emotions in an educational context are closely related to students’ and teachers’ performance, motivation, and well-being. In terms of students’ anxiety and enjoyment, the former was more thoroughly investigated [31] and studied by scholars in various fields, including those specialized in SLA within the framework of PP theories [32] and with an aim to explore how to achieve optimal learning [33,34,35]. Along with enjoyment and anxiety, boredom, as an emotion aroused during foreign language learning, has also been studied under the framework of flow theory in PP [36]. Apart from negative emotions, their positive counterparts such as love and empathy were also explored in regard to their relationship with language learners’ well-being [37,38].

Students’ enjoyment has been demonstrated as playing an essential role in promoting learners’ academic achievement and personal growth [39]. As for EFL students’ enjoyment, the issue has been studied in various contexts such as online collaborative or blended learning [40], private tutoring [41], overseas learning experience [42], and under the circumstances of specific domains of language skills such as listening, speaking, and reading [43]. Just as emotions can have a meditating effect on EFL learners’ language proficiency [44] and on their willingness to communicate [45], so does FLE which may also serve as a mediator facilitating higher motivation and better language proficiency [46]. Additionally, comparative studies on the emotions experienced by the different number and types of languages students acquire were also conducted. For instance, explorations were carried out investigating bilingual and multilingual learners’ FLE [47], the same individual’s FLE in learning first and foreign languages [48], and how different target languages can influence L2 students’ learning enjoyment [49].

Other factors that can shape students’ enjoyment include self-regulation, teacher behavior, and teacher support. First, what cannot be ignored is that students’ capability of self-regulating in the learning process, from the forethought phase to the self-reflection phase, can affect their emotions and may determine whether they can enjoy emotional stability or not [50]. Second, teachers were suggested to assist students to experience positive emotions [51,52], thereby strengthening the student–teacher relationship [53] and obtaining high-level student enjoyment [54]. Teacher behavior including the frequency of jokes teachers use has also been proven to have a significant impact on fostering a positive classroom environment [55]. Teachers’ and learners’ emotions and behaviors can mutually shape each other, as students’ enjoyment level can determine teacher engagement, which in turn might influence students’ performance and improvements in FL learning [56]. It is believed, therefore, that students and teachers can form a “joint-effort” when achieving and promoting FLE [57]. Third, teacher support has been perceived as an important factor determining students’ enjoyment [58], and such effect may appear to be even more obvious when examined in a longitudinal study [59]. Thoughts have also been expressed on how FLE can be determined by students’ perception of FLs and FL teachers [60].

Moreover, factors influencing students’ FLE may be characterized by both universal and situation-specific features. The relationship between FLE and gender, for example, may vary under different circumstances [61,62], and FLE can be shaped by external cultural elements [63]. Moreover, the level of FLE obtained by students reflects their autonomy and emotional intelligence, both of which indicate a learner’s ability to deal with challenging situations such as emergency and remote learning [64]. This capability of regulating emotions on various occasions indicates that the study of students’ FLE may be inseparable from ER. In this regard, further research designed specifically for examining the relationship between FLE, CL, and ER which can serve to further illuminate the issue of learners’ enjoyment in an FL learning context appears to be desirable.

Students’ ER: On top of discovering and analyzing the emotions experienced in an educational context, scholars generally contend that how people regulate emotions matters [65]. Hence, ER is regarded as a factor exerting positive influence on social adjustment [66]. That may explain why ER strategies have been applied during the learning process to mitigate negative emotions and promote positive emotions [67], both of which indicate the significance of regulating emotions in a learning context. What is more, students’ adaptation of ER strategies, which include suppression and reappraisal, have been studied across and within cultures [68], and it has been proven that students may implement different strategies to manage their emotions.

### 1.2. Research Hypotheses

Based on the above discussion, what can be seen is that it is timely and important to explore students’ FLE through the lens of positive psychology. Current studies, however, are far from satisfactory in revealing how EFL students’ interpersonal interactions such as PPS and PGI in a classroom climate may shape their FLE and how ER may impact the FLE of university EFL students. As EFL learners may endure considerable academic stress [69], thus experiencing continuously the feeling of anxiety, what seems necessary is to further investigate the relationship between FL learning and enjoyment, and in the case of this study, in a cooperative learning context. In order to explore the relationship between EFL learners’ FLE, CL, and ER, the following hypotheses were proposed:

1. ER strategies positively predict the FLE of university EFL learners.
2. Certain factor(s) which shape(s) CL in a classroom climate positively predict(s) the FLE of university EFL learners.

### 1.3. FLE, CL, and ER

Foreign language learning can be challenging, and FLE in this study refers to a positive emotion which can activate a positive state to use skills to meet these challenges, or using a term in positive psychology, it can stimulate a flow state [9]. Dewaele and MacIntyre [22] categorized FLE into FLE-social which relates to teachers, peers and environment and FLE-private which links to the personal cognition of enjoyment and a sense of accomplishment.

CL has been proven to have a positive effect on the outcome of schooling, and it can be defined as a component of social climate, which together with morphological climates such as school size and gender, form the two sides of classroom climate [24]. This study examined CL especially PGI and PPS in classroom climate experienced by students. PGI in the context of this study points to a conception that the goal of a team can be attained if and only if each member of the team is cooperatively linked to achieve the same goal. PPS refers to the support that members of a team provide for each other, reflecting the interpersonal relationship among students.

ER points to “the process by which individuals influence which emotions they have, when they have them and how they experience and express these emotions” [70]. In the context of this study, ER specifically entails students’ attempts to influence emotions in themselves. It also involves the ER strategies they adopt to achieve ER goals, which means that people may regulate their own emotions either for certain emotions being felt or certain purposes being achieved [71].

## 2. Materials and Methods

### 2.1. Context and Procedures

After obtaining ethical approval from the authors’ institution, and before the semester began in March 2022, 115 Chinese EFL students were invited to form teams of three or four members. In order to do so, the teacher, who is the first author of the paper, used the application Chaoxing Online Class (Chaoxing Xue Xi Tong), one of the major online teaching and learning platforms in China, and assigned a task of team building. As it was an English listening class where group study was rarely organized to assist teaching and learning, the students were informed that they would complete various assignments throughout the semester with their teammates. The students were invited through WeChat, one of the dominant social networking applications in China, to participate in this study and to complete a questionnaire anonymously. All the respondents participated on a voluntary basis and they were informed in the invitation letter of the purpose of the study and the confidentiality of the survey data. In the meantime, it had been stated in the invitation letter that they were encouraged to share their honest opinions to allow further improvements of both teaching quality and learning experience. All the data were collected using the data collection platform Credamo.

### 2.2. Participants and Context

Participants of this study were 115 first-year undergraduate EFL students who were invited to complete a questionnaire (all valid; 100%). The majority were female (N = 68; 59.1%). Age was between 18 to 20 (M = 18.69; SD = 0.65). Years of learning English ranged from 1 to 17 years (M = 11.94; SD = 2.40). The course was intensive in nature, as students were invited to work in groups to complete tasks calling for collaborative skills as well as language skills such as listening, speaking, and writing to ensure the attainment of a desirable learning experience.

### 2.3. Instruments

As the Chinese participants were English majors, a bilingual version questionnaire, presented both in English and Chinese, was provided for them (see Appendix A, the English version of the questionnaire). The students were encouraged to read the source text in English and if they came across words and expressions with which they were unfamiliar, they could refer to the corresponding Chinese version for more information. Three criteria were considered when designing the questionnaire of this survey. First, all the dimensions and items selected were highly relevant to the context of this research. Second, in order to guarantee the quality of the questionnaire, we had to control the length of finishing it within 10 min [72]. Third, we made sure that for each dimension measured in the questionnaire, the items used for measuring the dimensions can reflect its overall characteristics and only those that appeared to be redundant would be removed. For example, in the original positive goal interdependence (PGI) scale presented in the study of Ghaith et al. [24], it includes an item “When we work together in small groups, I have to find out what everyone else knows if I am going to be able to do the assignment”. This item, which has little connection with the study’s context, was removed from the scale.

*The FLE scale.* A shortened version of FLE scale developed from the 21-item initial version designed by Dewaele and MacIntyre [9] was used in the survey. The shortened version of the FLE scale was comprised of 10 items, covering enjoyment (e.g., “I enjoy it”), classroom climate (e.g., “It’s a positive environment”), collaboration (e.g., “We form a tight group”), sense of accomplishment (e.g., “In class, I feel proud of my accomplishments”), and teacher support (e.g., “The teacher is encouraging”). Participants were asked to rate their level of agreement on the descriptions of FLE on a five-point Likert scale ranging from 1 (“strongly disagree”) to 5 (“strongly agree”). The Cronbach’s alpha coefficient of the overall FLE scale was 0.85 which shows good reliability.

*ER questionnaire* (ERQ). The original version of ERQ is a 10-item measure of two emotion regulation strategies which are cognitive reappraisal and expressive suppression [73]. Cognitive reappraisal emphasizes how people through perceiving the situation from a different perspective manage to change the emotional impact that could have exerted on them, and as for expressive suppression, it refers to the inhibition of expressing one’s emotions [74]. The shortened 6-item ERQ adopted in this study consists of cognitive reappraisal (3 items; e.g., “When I want to feel more positive emotion, I change the way I’m thinking about the situation”) and expressive suppression (3 items; e.g., “When I am feeling negative emotions, I make sure not to express them”). Participants were asked to rate their level of agreement on the descriptions of ERQ on a five-point Likert scale ranging from 1 (“strongly disagree”) to 5 (“strongly agree”). The Cronbach’s alpha coefficient of the overall FLE scale was 0.70 which shows adequate reliability.

*PGI scale* and *PPS scale*. Both are derived from the subscales of a modified 48-item version of The Classroom Life Measure [24]. The shortened PGI scale adopted in this study contains 5 items, instead of 6 as included in the modified version provided by Ghaith et al. [24] (e.g., “When we work together in small groups, everyone’s ideas are needed if we are going to be successful”). The shortened PPS scale has 4 items, in which 1 item was removed compared to the version provided in Ghaith et. al. Participants were asked to rate their level of agreement on the descriptions of PGI and PPS on a five-point Likert scale ranging from 1 (“strongly disagree”) to 5 (“strongly agree”). The Cronbach’s alpha coefficient of the overall PGI scale and PPS scale were 0.85 and 0.82 respectively which shows good reliability.

**Figure 1:**
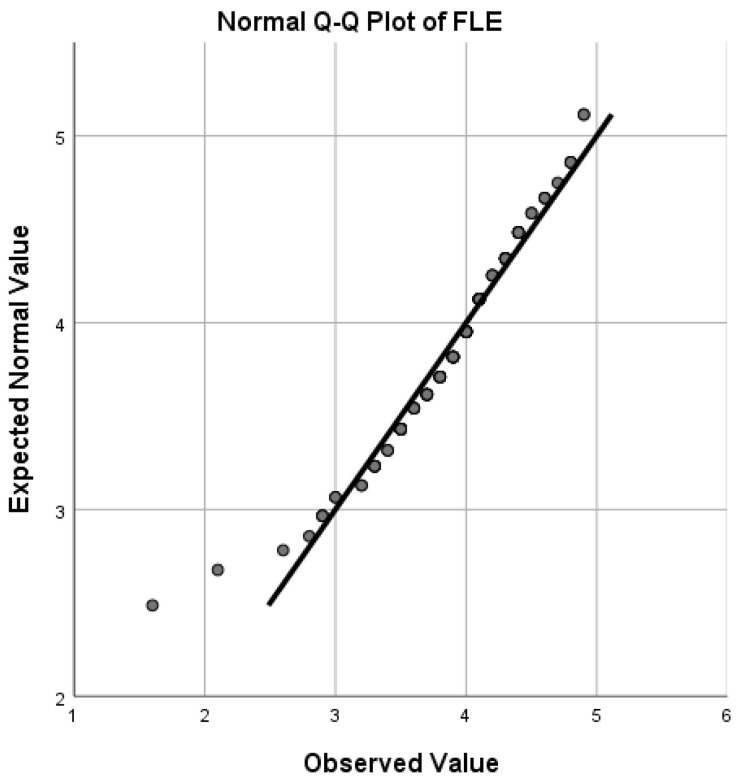
Quantile-quantile (Q-Q) plot for distribution of FLE scores.

**Figure 2:**
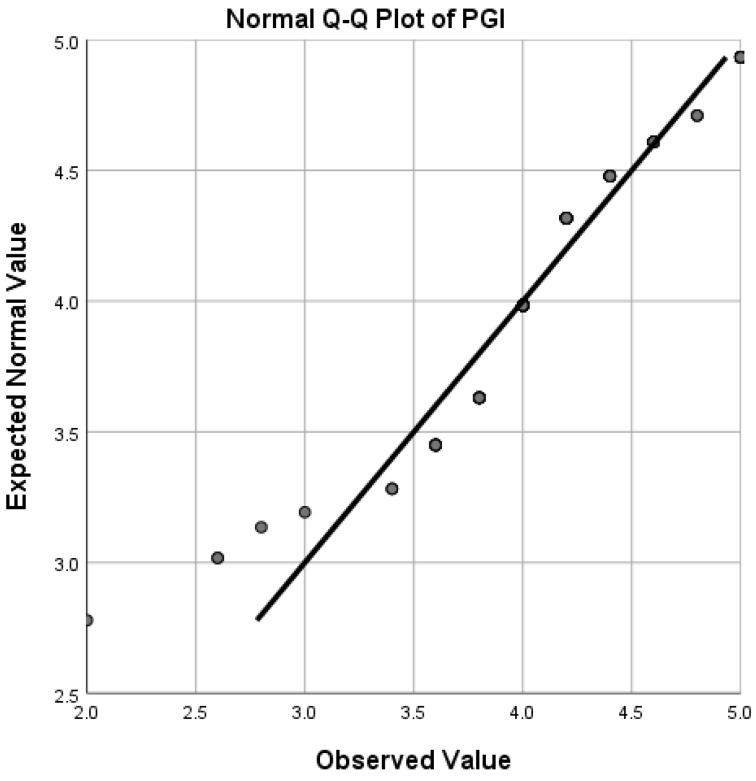
Quantile-quantile (Q-Q) plot for distribution of PGI scores.

**Figure 3:**
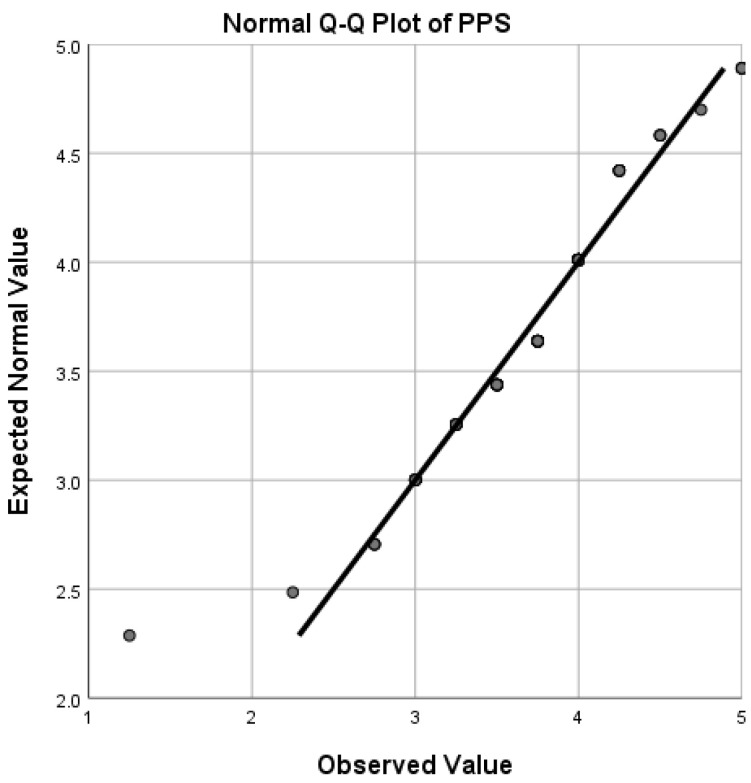
Quantile-quantile (Q-Q) plot for distribution of PPS scores.

**Figure 4:**
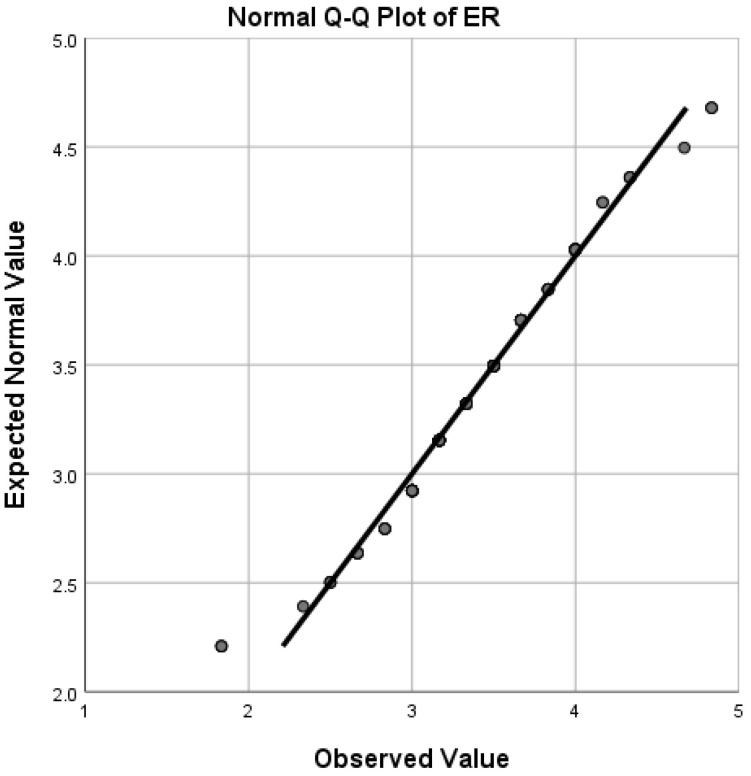
Quantile-quantile (Q-Q) plot for distribution of ER scores.

Q-Q (quantile-quantile) plots (see Figure 1, Figure 2, Figure 3 and Figure 4) showed that FLE, PPS, PGI, and ER scores followed a normal distribution reasonably well, except for the extreme tail, thus enabling us to choose *t*-tests and Pearson correlations which are regarded as powerful parametric tests [64].

### 2.4. Data Analysis

This study employed a cross-sectional, quantitative design. SPSS 26.0 was adopted to conduct data analysis in the present study. First, Harman’s single-factor test was performed to assess the common method bias. Second, the reliability of each scale used was tested by Cronbach’s Alpha and only those that showed good reliability were selected and used. Third, descriptive statistics were generated including mean and standard deviation. Fourth, Pearson’s correlation analysis was performed among all the variables to analyze the correlation between FLE and ER as well as between FLE and the factors which shape CL. Fifth, we ran multiple linear regression analysis to investigate and determine the predictors of FLE.

## 3. Results

The results will be reported in two sections, organized by the research hypotheses. In this study, Harman’s single-factor test was used to assess the common method bias. It was found that a single factor accounted for 31.37% of the variance which is below the critical value of 40% [75], indicating that there was no serious common method bias in the current study.

### 3.1. ER Strategies as the Predictor of FLE

Under the circumstances of cooperative learning in an English listening class, the results demonstrated that FLE was positively correlated with ER (γ = 0.35, *p* < 0.01), as it is shown in Table 1. ER also served as a predictor, showing a significant influence on FLE (β = 0.22, t = 2.94, *p* = 0.004).

### 3.2. PGI and PPS: Forms of Cooperation That Can Predict FLE

In the classroom life measure scale, there are nine subscales [24], and after testing the reliability coefficient of the subscales, it was discovered that FLE was positively correlated with both PGI (γ = 0.53, *p* < 0.01) and PPS (γ = 0.76, *p* < 0.01), which is presented in Table 1. As the two elements that shape cooperation in a classroom climate, PGI and PPS also showed positive correlation with each other. This suggests that EFL students who are more capable of cooperation both in achieving tasks and in supporting other team members, tend to have high FLE in a classroom environment.

**Figure 5:**
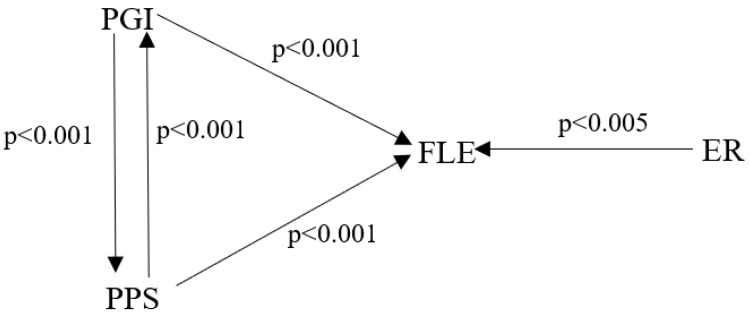
Regression model of university EFL students’ FLE.

According to the results of regression analysis presented in Table 2, PGI significantly and positively influenced students’ FLE (β = 0.32, t = 3.80, *p* < 0.001), and PPS was also a significant predicator of FLE (β = 0.33, t = 3.81, *p* < 0.001). The three predictors accounted for a significant amount of variance in EFL students’ FLE. The overall regression model is significant (see Figure 5).

## 4. Discussion

The purpose of this study was to gain an insight into the factors that could shape university EFL students’ FLE. It found out that students with good collaboration skills and ER abilities were more likely to find enjoyment during FL learning. The main findings will be reported in two parts. The first focuses on the specific context of SLA especially the significant influence of ER on FLE. The second part concentrates on the significance of peer support particularly the intertwined relationship between peers’ interdependence, achievement of common goals, and enjoyment.

First, this study discovered the importance of ER in shaping university EFL students’ FLE. The finding is in line with previous research focusing on language learners’ enjoyment and ER [40]. Similar findings were also reported among teachers whose FLE could be shaped by the ER strategies they adopted [76]. Along with other positive emotions such as love, pride, and hope which can lead to enjoyment [77], this study proved a particular relevance between ER and FLE. Learning a foreign language can be challenging, and that also sheds light on why a vast majority of scholarship studying emotion experiences of FL learners focused on the anxiety endured by FL students. This study, as part and parcel of the positive psychology movement in SLA studies [59], highlights the significance of ER for EFL students’ enjoyment generation.

ERQ in this study measured two ER strategies which were expressive suppression (M = 2.94; SD = 0.71) and cognitive reappraisal (M = 3.88; SD = 0.63). This result indicated that students tended to hold a more preferable attitude towards cognitive reappraisal, as they would like to change the emotion impact [74] by perceiving a situation from a different and probably more positive and promising perspective. It is not surprising, therefore, that ER investigated in this study could positively predict FLE. Another indication in this regard is that EFL students, usually with a tight schedule and demanding tasks on developing comprehensive language and intercultural communication skills, might consciously develop their ER abilities especially reappraisal for the aim of achieving enjoyment of FL learning. This is also in line with the previous study which discovered a positive impact of emotional engagement on language learning, as for proactive and reluctant learners alike, if they can be emotionally engaged in the course, they would be more likely to achieve higher motivation in an FL learning context [78].

Second, interdependence and support among team members played a pivotal role in determining university EFL learners’ FLE. Concerning the specific context of this study, which happened in an English listening class, although much attention has been paid to how students’ language skills and critical thinking ability can be developed [79,80], little research has been conducted to explore how CL can influence university EFL learners’ FLE in a listening class.

This study confirms previous research findings that supportive relationships can improve students’ learning experiences [64] and impact their learning motivation [81]. More specifically, it paid attention to what forms of CL could determine students’ FLE. The corresponding discovery confirmed the role of peer support in predicting enjoyment [82], a discovery that is in line with that of a previous study which argues that peer support can contribute to positive emotions [83]. What seems special about the present study is that it highlighted the significance of peer support rather than predominantly focusing on teacher support in achieving FLE. In previous research, teachers’ style of instruction and the support they offered attracted attention and was thoroughly investigated [84,85]. Teacher-related variables’ prediction of FLE has also been thoroughly discussed [52,86,87]. However, under the circumstances of CL, more attention should be paid to teammates or peers from the same class, especially to how PGI and PPS may predict and to what extent they can predict the FLE of students. The result which showed a positive and significant prediction of PGI and PPS on FLE is understandable. This is because if all the members of the same group can act interdependently and share their ideas for achieving the same goal, they are likely to voluntarily support each other. The sense of interdependence felt during collaboration and the sense of accomplishment displayed after completing the task could help students generate an enjoyable FL learning experience.

Moreover, this result indicated that PPS in a collaborative context could be crucial. Students, whenever cooperating, need to provide each other with support if they would like the task to proceed smoothly and if they want to achieve a common goal. This study, in contrast to the discovery reported in Ghaith et al. [24], found a significant relationship between PPS and PGI. This can be explained by the fact that in a CL context, apart from team members’ individual academic competence, what appears to be of utmost importance is the interpersonal relationship between learners and the degree of their interdependence in terms of achieving the same goal.

## 5. Implications and Limitations

### 5.1. Theoretical and Practical Implications

The theoretical implication of this study is twofold. From the perspective of students’ FLE, it indicated that ER, especially reappraisal, might play a pivotal role in determining students’ enjoyment in FL learning. As reappraisal has been proven to play a mediating role which can help boost enjoyment in an online teaching context [23], this study further enriched the implication of ER theory by indicating the significance of reappraisal in determining EFL students’ enjoyment. Furthermore, it indicated that from an interpersonal perspective, positive collaboration might shape students’ enjoyment in an FL learning context. As implied in previous research, CL was positively correlated with PPS [88], and this study further implied that both PPS and PGI in a CL context could determine EFL learners’ enjoyment, and therefore highlights the significance of the specific dimensions of CL which can influence FLE.

The study also offers implications for both educational practitioners and EFL learners. As ER, PPS, and PGI are the determining factors shaping FLE, EFL teachers should encourage students to use reappraisal whenever necessary both in the process of FL learning and in the course of CL. In terms of CL, what needs to be highlighted if EFL teachers design activities involving teamwork is that they can consciously cultivate an atmosphere of interdependence both academically and interpersonally between students. For EFL students who may face considerable academic stress throughout the university years, the application of ER strategies, particularly reappraisal, may enable them to conduct productive cooperation and hopefully facilitate them to achieve considerable improvements enjoyably and positively.

### 5.2. Limitations

This study is not without limitations. First, the data were collected using a questionnaire survey, and future studies may adopt a mixed-method research design to explore the FLE in a cooperative learning context. Second, the sample size of this study was relatively small as all the students invited were attendees of the listening class taught by the first author of the article. In addition, as emotions can change over time, it will be interesting to conduct longitudinal research with control and experimental groups. Therefore, further studies can be designed with larger sample size and consider exploring the change of students’ FLE during cooperative learning over a period of time.

## 6. Conclusions

The results of this study suggested that EFL students’ FLE could be predicted by PGI and PPS, two factors which are part and parcel of CL in an educational context, and by ER, the strategies of which may be adopted to influence and change learners’ own emotions. The current findings suggested that students who were able to perceive challenges from a positive perspective and to collaborate proactively with teammates were more likely to achieve FLE. The findings of this research may be useful for both teachers and students. Teachers may share with students the concepts of ER strategies and the significance of positive cooperation to facilitate students’ realization of enjoyable FL learning experience. Students, in a similar way, can be instructed by teachers to use ER strategies to regulate their emotions and to boost the sense of mutual support and interdependence during cooperative learning, thus becoming happier and more proficient FL learners.

## Figures and Tables

**Table 1:** Descriptive statistics and correlation analysis (N = 115).

	M(SD)	1	2	3	4
1. FLE	3.86 (0.54)	1			
2. PGI	4.10 (0.52)	0.53 **	1		
3. PPS	3.72 (0.56)	0.57 **	0.54 **	1	
4. ER	3.41 (0.55)	0.35 **	0.12	0.27 **	1

** *p* < 0.01.

**Table 2:** Linear regression analysis for variables predicting FLE.

Predictor	β	B	t	*p*
PGI	0.32	0.34	3.80	0.000 ***
PPS	0.33	0.32	3.81	0.000 ***
ER	0.22	0.22	2.94	0.004 **
Model Index				
R	0.66			
R^2^	0.44			
Adjusted R^2^	0.42			
F	28.443			

** *p* < 0.01, *** *p* < 0.001.

## Data Availability

The datasets used and/or analyzed during the current study are available from the corresponding author upon reasonable request.

## Appendix A

A Survey of Foreign Language Enjoyment in a university English Class.

**Table A1:** Here are some statements about foreign language enjoyment. To what extent do you agree with the following statements?

		Strongly Disagree	Disagree	Neither Agree nor Disagree	Agree	Strongly Agree
1	I do not get bored.	1	2	3	4	5
2	We form a tight group.	1	2	3	4	5
3	I enjoy it.	1	2	3	4	5
4	It is a positive environment.	1	2	3	4	5
5	I have learned interesting things.	1	2	3	4	5
6	It is fun.	1	2	3	4	5
7	The teacher is friendly.	1	2	3	4	5
8	The teacher is supportive.	1	2	3	4	5
9	In class, I feel proud of my accomplishments.	1	2	3	4	5
10	The teacher is encouraging.	1	2	3	4	5

**Table A2:** Here are some statements about teamwork. To what extent do you agree with the following statements?

		Strongly Disagree	Disagree	Neither Agree nor Disagree	Agree	Strongly Agree
1	When we work together in small groups, we try to make sure that everyone in the group learns the assigned material.	1	2	3	4	5
2	When we work together in small groups, our job is not finished until everyone in the group has completed the assignment.	1	2	3	4	5
3	When we work together in small groups, everyone in the group has to share materials with other team members.	1	2	3	4	5
4	When we work together in small groups, everyone’s ideas are needed if we are going to be successful.	1	2	3	4	5
5	When we work together in small groups, we all receive bonus points if everyone scores above a certain criterion.	1	2	3	4	5

**Table A3:** Here are some statements about emotion regulation strategies). To what extent do you agree with the following statements?

		Strongly Disagree	Disagree	Neither Agree nor Disagree	Agree	Strongly Agree
1	When I want to feel a more positive emotion, I change the way I am thinking about the situation.	1	2	3	4	5
2	I keep my emotions to myself.	1	2	3	4	5
3	When I am feeling negative emotions, I make sure not to express them.	1	2	3	4	5
4	When I am faced with a stressful situation, I make myself think about it in a way that helps me stay calm.	1	2	3	4	5
5	When I want to feel less negative emotion (such as sadness or anger), I change what I am thinking about.	1	2	3	4	5
6	When I am feeling positive emotions, I am careful not to express them.	1	2	3	4	5
7	When I am feeling positive emotions, I am careful not to express them.					

**Table A4:** Here are some statements about peer support. To what extent do you agree with the following statements?

		Strongly Disagree	Disagree	Neither Agree nor Disagree	Agree	Strongly Agree
1	My teammates in this class think it is important for them to be my friends.	1	2	3	4	5
2	My teammates in this class like me the way I am.	1	2	3	4	5
3	My teammates in this class care about my feelings.	1	2	3	4	5
4	My teammates in this class like me as much as they like other members in the team.	1	2	3	4	5
